# The Bookworld of Medicine and Science

**Published:** 1904-07-02

**Authors:** 


### The Bookworld of Medicine and Science.

Physiology and Pathology of the Urine. By J. Dixon Mann, M.D., F.R.C.P. (London: Griffin and Co., 1904. Pages 272. Price 8s. 6d. net.)

Any book written under the name of the above author is sure to meet with a warm welcome and be read with respect, and this volume in no way disappoints our expectations. As stated in the preface, the book is primarily intended to serve as a clinical guide in the diagnosis and treatment of disease, but in reality it has much wider bearings, and though the various processes which are suitable to the clinical laboratory are fully described, deeper levels are touched which are only possible for the skilled chemical pathologist. Looked at from this point of view, the book attains a high standard, and though the average student could, with a little trouble, find in it all that is necessary for him to know for purposes of examinations, the book is better fitted for the more advanced student who is making the urine a field for special research. As a book of reference also to the practitioner, it must prove of great value, as careful directions are given for the detection of many unusual substances, various pigments and colouring matters, etc., which are too seldom met with for him to be very familiar with. If there is a weak point to be found, it is, we think, in the section dealing with micro-organisms in urine. Of late years it has been shown that the recognition of the presence and the nature of bacteria in urine, and the changes they produce in the urine, is frequently of very great clinical importance, and at any rate it is a subject deserving of considerable attention in a book dealing with the pathology of urine.

Medical Monograph Series. No. X. Cleft-Palate and Hare-Lip. By Edmund Owen, M.B., F.R.C.S. (London: Baillière, Tindall and Cox. Pp. 111. Forty-one Illustrations. Price 2s. 6d. net.)

This little book does not aim at being a symposium of all that is known or has been written on cleft palate; it sets forth the personal experience of a practical surgeon, and in this lies its great merit. Too often the modern scientific text-book wearies out the reader with dry, laboriously-collected extracts from the writings of numerous authorities. Mr. Owen commences with a short account of the development of the palate and lip, and goes on to consider the preparation for and the details of operation, and the after treatment of the patient, and includes a large amount of thoroughly practical instruction on these points. The most striking feature in the work is Mr. Owen's endorsement of Brophy's operation for cleft palate. The latter surgeon operates on cleft palate in early infancy leaving an associated hare-lip for treatment later on, and the essential feature of his procedure is the bringing together of the margins of the cleft by firm pressure over the malar bones, or, if this be not sufficient, by cutting through the maxillary bones above the palate and then pressing the two halves of the palate together and retaining them in position with wire sutures.

A Manual for the Sick and Sorrowful. Arranged by E. S. L., Joint Compiler of "The Children's Morning Text-book." Second Edition. (London: Elliot Stock, 62 Paternoster Row, E.C. 1904. Price 3s.)

That this manual is well adapted to the purpose for which it has been compiled is evidenced by the fact that a second edition has been called for within fifteen months of the publication of the first. The selections are well chosen, judiciously brief, and, apart from their special use in the sick-room and the house of mourning, are interesting as giving the thoughts of a number of the most prominent churchmen, living and recently dead, regarding sickness, suffering and death. The book should find many readers.

